# Breast Cancer Incidence and Groundwater Contamination in Southwestern Ohio: An Ecological Analysis of Communities Overlying the Great Miami Buried Valley Aquifer

**DOI:** 10.7759/cureus.113584

**Published:** 2026-07-29

**Authors:** Rebecca Glaser

**Affiliations:** 1 Surgery, Boonshoft School of Medicine, Wright State University, Dayton, USA

**Keywords:** breast cancer, cancer surveillance, ecological study, environmental epidemiology, environmental justice, groundwater contamination, ohio, pfas

## Abstract

Background

Regional variation in breast cancer incidence may reflect environmental factors in addition to established risk factors. Southwestern Ohio overlies the Great Miami Buried Valley Aquifer (GMBVA), a sole-source aquifer that has experienced long-term contamination by volatile organic compounds (VOCs) and documented detections of per- and polyfluoroalkyl substances (PFAS). This ecological study examined breast cancer incidence in counties overlying portions of the GMBVA with the most extensively documented groundwater contamination. Contamination was defined at the county level using publicly available environmental records, including federal Superfund site designations, US Geological Survey (USGS) PFAS detections, and Department of Defense inventories of PFAS response sites. County location over the aquifer was used strictly as a contextual population-level proxy rather than as a measure of individual exposure.

Methods

Age-adjusted invasive female breast cancer incidence rates for 2017-2021 and 2018-2022 were obtained from the Surveillance, Epidemiology, and End Results (SEER) Program, the National Cancer Institute State Cancer Profiles, and the Ohio Cancer Incidence Surveillance System for all ages, women aged 50 years and older, and women aged 65 years and older. Rates were compared with the corresponding Ohio statewide and US reference rates from the same reporting periods. Age-adjusted incidence rate ratios (IRRs) were computed by dividing the Montgomery County age-adjusted rate by the corresponding Ohio statewide and US reference rates, with 95% CIs derived by log transformation of the published rate standard errors. Publicly available data from the USGS, US Environmental Protection Agency (EPA), Ohio EPA, Miami Conservancy District, and US Air Force were used to characterize groundwater contamination and remediation in the region.

Results

Ohio’s statewide rate was 132.3 per 100,000 (95% CI: 131.2-133.4), compared with 130.8 nationally (95% CI: 129.9-131.7). Montgomery County, which overlies heavily contaminated sections of the GMBVA, had a rate of 141.4 per 100,000 (95% CI: 135.7-147.3), approximately 8% above the national benchmark (standardized incidence ratio (SIR): 1.08, 95% CI: 1.04-1.13 vs. the US; SIR: 1.07, 95% CI: 1.02-1.11 vs. Ohio). Relative differences appeared somewhat larger in the older age strata (SIRs: approximately 1.08-1.13), although the ecological design precludes interpreting age as a proxy for cumulative exposure duration or timing. PFAS were detected in 11 of 23 GMBVA wells (USGS, 2019-2020); VOC concentrations at Montgomery County Superfund sites historically exceeded maximum contaminant levels by orders of magnitude. Descriptively, several core industrialized counties appeared to have higher incidence rates than less-industrialized counties in the region.

Conclusions

This ecological, hypothesis-generating analysis identified descriptive county-level patterns of modestly elevated age-adjusted invasive female breast cancer incidence in parts of southwestern Ohio overlying historically contaminated portions of the GMBVA. The ecological design, severe potential for exposure misclassification, lack of adjustment for confounding, qualitative rather than formal spatial comparison, and inability to assess residential history, water source, exposure timing, or latency preclude causal inference or individual-level interpretation. These findings should be interpreted strictly as contextual and descriptive.

## Introduction

Breast cancer is the most commonly diagnosed invasive cancer among women in the United States (US), and its incidence varies markedly across counties and states [1,2]. Although established risk factors, including age, reproductive history, family history, hormonal exposures, alcohol use, obesity, and screening intensity, explain much of this variation, regional differences may also reflect environmental conditions, including chronic, low-level exposure to drinking-water contaminants [1]. Volatile organic compounds (VOCs), such as trichloroethylene (TCE) and tetrachloroethylene (PCE), as well as per- and polyfluoroalkyl substances (PFAS), are widely detected industrial contaminants with documented or suspected carcinogenic and endocrine-disrupting properties [1,3-5].

Southwestern Ohio overlies the Great Miami Buried Valley Aquifer (GMBVA), a federally designated sole-source aquifer that supplies drinking water to approximately 2.3 million residents and provides 97% of the region’s public water supply [6,7]. The aquifer extends across roughly 15 counties drained by the Great Miami River and its tributaries, an area of approximately 5,880 square miles [6]. Because of its high transmissivity and shallow water table, the GMBVA is unusually vulnerable to surface and near-surface contamination, and it has experienced long-term impacts from industrial solvents and PFAS originating from manufacturing, fire-training, and military operations [6,8-10].

Regional variation in breast cancer incidence has been noted in southwestern Ohio, including in Montgomery County, coinciding with areas of documented long-term groundwater contamination from industrial and military sources. This ecological analysis was undertaken to descriptively examine county-level, age-adjusted breast cancer incidence in relation to publicly available data on GMBVA contamination.

Previous epidemiologic investigations have reported associations between PCE-contaminated drinking water and breast cancer in Cape Cod, Massachusetts [11-13], and have raised concerns about solvent exposure at Camp Lejeune, North Carolina [14]. Meta-analyses of PFAS and breast cancer suggest small, imprecise positive associations for specific compounds and exposure windows but no consistent overall effect [15,16]. To the author’s knowledge, however, no prior study has examined breast cancer incidence in counties overlying the GMBVA in relation to documented VOC and PFAS contamination of this sole-source aquifer.

The present ecological, hypothesis-generating study addresses this gap. To the author’s knowledge, and based on targeted PubMed and Google Scholar searches using terms combining “Great Miami” or “Miami Valley” with “breast cancer” and “groundwater,” “PFAS,” or “solvent” (searches conducted during 2025-2026), this study is among the first analyses to integrate county-level breast cancer incidence data from the Surveillance, Epidemiology, and End Results (SEER) Program, the National Cancer Institute State Cancer Profiles, and the Ohio Cancer Incidence Surveillance System with publicly available contamination records from the U.S. Geological Survey (USGS), U.S. Environmental Protection Agency (EPA), Ohio Environmental Protection Agency (Ohio EPA), and Miami Conservancy District for the GMBVA region.

## Materials and methods

Study area

Geographic Setting and Topography

The study area encompasses the 15 southwestern Ohio counties that overlie the GMBVA, as delineated by the USGS and Miami Conservancy District [6,7]: Montgomery, Butler, Miami, Greene, Warren, Hamilton, Clark, Champaign, Shelby, Preble, Darke, Logan, Auglaize, Mercer, and Hancock counties [6,7].The region is drained by the Great Miami River, the Little Miami River, and the Whitewater River, which together drain approximately 5,880 square miles of Ohio, with an additional 1,329 square miles of the Whitewater River watershed in southeastern Indiana [6]. The topography is gently rolling, with broad alluvial valleys incised into Ordovician shales and Devonian-Silurian carbonate bedrock; elevations in the city of Dayton are approximately 225 m (740 ft) above sea level.

Climate

Southwestern Ohio has a humid continental climate (Köppen Dfa), with hot, humid summers and cold winters [17]. The mean annual temperature in Dayton is approximately 11-12 °C (52 °F), with average daily high temperatures in July of approximately 30 °C (85 °F) and average daily low temperatures in January of approximately -8 °C (18 °F). Mean annual precipitation is approximately 1,040-1,050 mm (41 in) and is distributed relatively evenly throughout the year, with a spring maximum (approximately 115 mm in May) and a late-winter minimum (approximately 60 mm in February); mean annual snowfall is approximately 590 mm (23 in) [17]. Rapid infiltration and short residence times in the unsaturated zone facilitate the downward migration of soluble contaminants, including chlorinated solvents and short-chain PFAS, into the underlying glacial outwash aquifer.

Hydrogeology of the GMBVA

The GMBVA is a glacial outwash and alluvial sand-and-gravel aquifer that fills a system of buried Pleistocene bedrock valleys [6]. Aquifer sediments range from 0 to nearly 400 ft in thickness, most commonly 150-200 ft, and well yields commonly exceed 1,000 gallons per minute at municipal supply wells [6]. Hydraulic conductivity ranges from approximately 0.33 to 2,500 ft/d in the most permeable valley-fill deposits, and groundwater recharge through coarse-grained surficial deposits averages 6-15 inches per year, substantially greater than recharge through adjacent fine-grained till areas (<3 to approximately 5 inches per year) [6]. Groundwater-age estimates derived from tritium-helium-3 data indicate apparent residence times ranging from a few months to several decades, with the majority of sampled wells having been recharged during the period of widespread PFAS and chlorinated-solvent use (1947 to the present) [6].

These properties, high permeability, a shallow water table, rapid recharge, and induced infiltration from streambeds where pumping reverses the normal direction of flow, make the GMBVA highly vulnerable to surface-derived contamination.

Socioeconomic Characteristics, Water Use, and Pollutant Sources

Montgomery County, the most populous county overlying the GMBVA, had a 2020 census population of 537,309 and an estimated 2024 population of approximately 535,000-542,000, with a median household income of approximately $66,000 and an estimated poverty rate of approximately 15% [18,19]. The county includes the city of Dayton, with a population of approximately 137,000, one of Ohio’s largest urban centers, and a constellation of older industrial suburbs, including Kettering, Riverside, Huber Heights, Miamisburg, and West Carrollton. The surrounding counties (Butler, Greene, Warren, and Miami) include both densely populated suburban municipalities and substantial areas of agricultural land.

The Dayton metropolitan area was historically a national center of heavy manufacturing, automotive parts production, and machine tooling and is currently dominated by aerospace and defense research (Wright-Patterson Air Force Base, approximately 21,000 employees), health care (Premier Health and Kettering Health), advanced manufacturing, and logistics [18]. Agricultural land surrounding the urban core supports row crops, including corn and soybeans, as well as dairy and livestock production. Approximately 97% of regional water demand is met by the GMBVA, including municipal supplies, industrial process water, commercial uses, and agricultural irrigation [6,7]. Documented sources of groundwater contamination include legacy chlorinated-solvent releases from automotive, aerospace, and dry-cleaning operations; aqueous film-forming foam (AFFF) use at Wright-Patterson Air Force Base; contamination associated with historical nuclear weapons-component manufacturing at the former Mound Plant in Miamisburg; landfill leachate; combined sewer overflows; and agricultural runoff [8-10,20,21]. Several of these facilities or source areas have been designated as federal Superfund sites or active Department of Defense PFAS-response sites [9,10,20,21].

Study design

The GMBVA region is also the geographic catchment area for the authors’ clinical practice (Millennium Wellness Center, Dayton, Ohio), which predominantly serves patients from Montgomery, Greene, and Warren counties. The authors noted that age-adjusted invasive breast cancer incidence in Montgomery County was consistently elevated relative to both statewide Ohio and U.S. reference rates from SEER, which motivated the present ecological analysis of the aquifer region. Importantly, the a priori designation of focal versus comparator counties used in this analysis was based solely on independently documented environmental criteria (specified below) and not on prior knowledge of county-level incidence rates or the geographic distribution of the authors’ clinical practice; Butler and Miami counties were included in the focal group on environmental grounds despite falling outside the practice’s primary catchment area, and Warren County was treated as a comparator despite being within the catchment area.

This county-level, hypothesis-generating ecological study examined invasive female breast cancer incidence in relation to regional groundwater contamination in southwestern Ohio. Montgomery County and the adjacent core-watershed counties (Butler, Miami, and Greene) were designated a priori as the focal comparison area based on prespecified, publicly documented contamination criteria: the presence of one or more federal Superfund sites with chlorinated-solvent groundwater plumes (U.S. EPA Superfund site profiles); documented PFAS detections in the USGS 2019-2020 GMBVA well survey; or listing as an active U.S. Department of Defense PFAS-response site. Counties overlying the GMBVA that did not meet any of these criteria were treated descriptively as comparators with a lower documented contamination context. A county’s location over the aquifer and the presence of these documented contamination features were used strictly as contextual, population-level proxies for potential exposure and not as individual-level exposure measures [6,7,9,10].

Outcome Data Sources

Age-adjusted invasive female breast cancer incidence rates and corresponding 95% CIs were obtained from the SEER Program, the National Cancer Institute State Cancer Profiles, and the Ohio Cancer Incidence Surveillance System for the periods 2017-2021 and 2018-2022 [22-24]. Different reporting periods were used because these publicly available surveillance systems release rates according to nonidentical schedules. At the time of the analysis, the most recent Ohio Cancer Incidence Surveillance System release covered 2017-2021, whereas national SEER data and the State Cancer Profiles county-level data for Montgomery County covered 2018-2022. Because the two periods differed by only one calendar year at each end and overlapped for four years (2018-2021), and because breast cancer incidence in these populations had been relatively stable over this interval, the resulting comparison was considered unlikely to have been materially affected; nevertheless, this partial nonoverlap was noted as a limitation. Rates were expressed per 100,000 women and age-adjusted to the 2000 U.S. standard population. Rates were extracted for all ages combined and for the subgroups of women aged 50 years and older and 65 years and older.

Environmental Contamination and Contextual Exposure Data

Publicly available information on groundwater contamination, remediation activities, and contaminant occurrence was compiled from USGS Scientific Investigations Reports [6], US EPA Superfund site profiles [9,20,21], Ohio EPA watershed reports [8], Miami Conservancy District technical reports [7], and US Air Force monitoring updates for Wright-Patterson Air Force Base [10,25]. These sources were used to characterize the county-level contamination context and relative exposure potential, particularly for PFAS and chlorinated solvents (TCE and PCE).

Variables and Comparisons

The primary outcome was age-adjusted invasive female breast cancer incidence. Rates were compared for all ages combined and for the subgroups of women aged 50 years and older and 65 years and older. The ≥50 and ≥65 age strata were selected a priori because they align with the age groups for which the National Cancer Institute State Cancer Profiles and Ohio Cancer Incidence Surveillance System publish separate rates and CIs, because they encompass the age range in which the majority of invasive female breast cancers are diagnosed and mammographic screening is routinely recommended, and because they permit examination of whether patterns differ among older women, in whom cumulative background risk is higher. These strata were not intended to represent a longer duration of exposure or specific exposure windows because the available data did not include residential history, water source over time, or exposure timing. County-level contamination context was treated as a crude population-level proxy for potential exposure rather than an individual-level exposure measure; no data on individual residential history, water source, or exposure timing were available.

Statistical Analysis

The analysis was descriptive and comparative. Percentage differences between county, state, and national incidence rates were calculated, and 95% CIs were examined to assess the precision of the observed differences. Because the data required to calculate a conventional indirectly standardized incidence ratio (SIR), observed cases divided by expected cases derived from stratum-specific reference rates applied to the county’s population structure, were not uniformly available across the three surveillance systems, we instead calculated the ratio of the Montgomery County age-adjusted rate (2000 U.S. standard) to the corresponding age-adjusted reference rates for Ohio and the United States (SEER). This measure is an age-adjusted incidence rate ratio and is not mathematically equivalent to a conventional indirectly standardized SIR, even when both rates are age-adjusted to a common standard population. It is reported here as an “SIR” solely for continuity with prior descriptive cancer-surveillance literature; readers should interpret the measure as an age-adjusted incidence rate ratio. The 95% CIs for each reported rate ratio were derived analytically on the log scale using standard errors estimated from the published rates and their 95% CIs [26,27]. Geographic correspondence between elevated incidence and areas of documented contamination was evaluated qualitatively. Because the study relied on aggregated public data, no individual-level covariates or multivariable regression models were included. Review by an institutional review board was not required.

## Results

Statewide and national incidence comparisons

Ohio’s age-adjusted invasive female breast cancer incidence rate was 132.3 per 100,000 women (95% CI: 131.2-133.4) during 2017-2021 [23]. The national SEER incidence rate for 2018-2022 was 130.8 per 100,000 women (95% CI: 129.9-131.7) [22]. Thus, Ohio’s rate was approximately 1.1% above the national benchmark.

Montgomery County Findings

**Figure 1:**
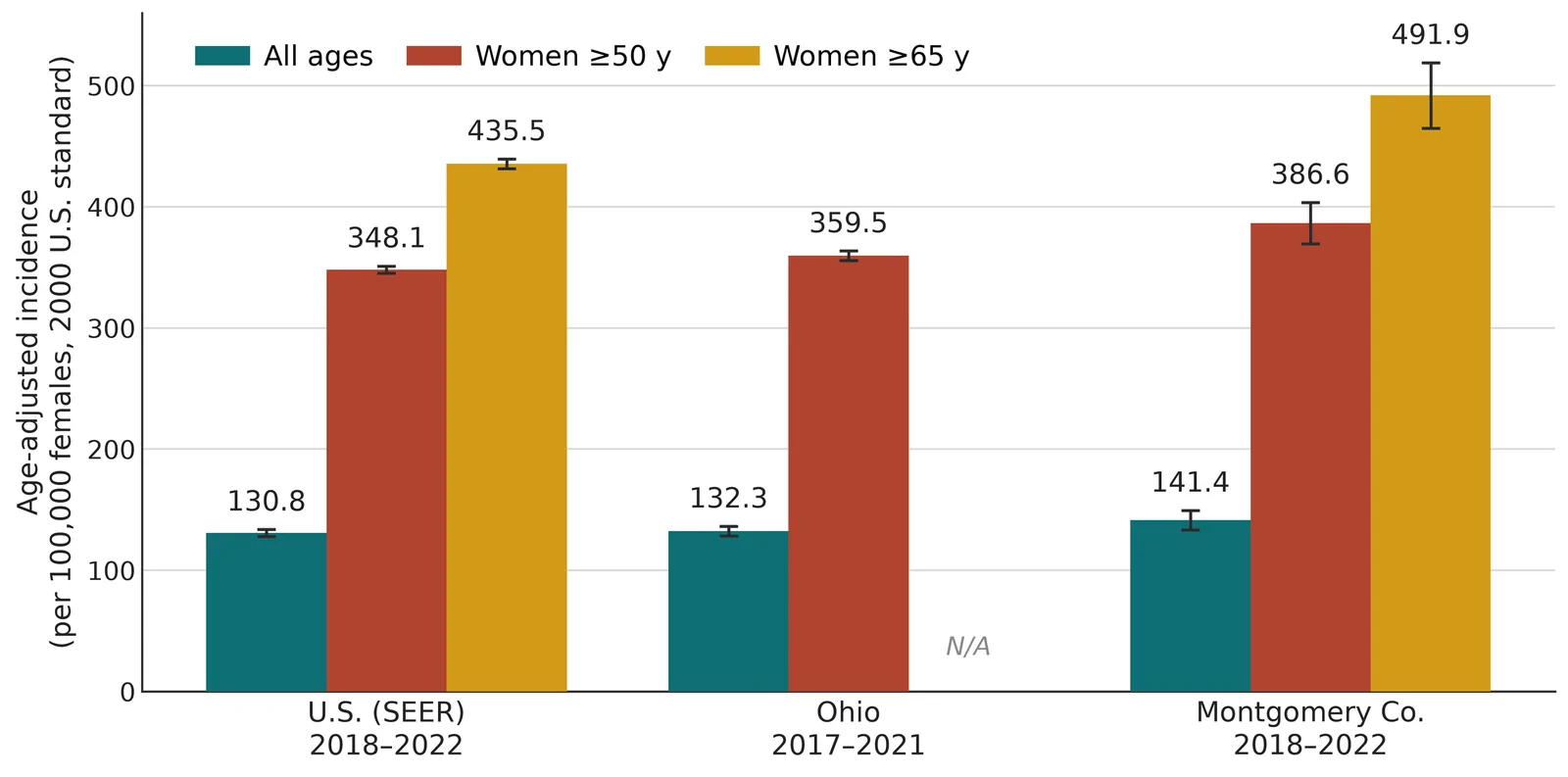
Age-adjusted invasive female breast cancer incidence rates. Age-adjusted invasive female breast cancer incidence rates per 100,000 females, standardized to the 2000 United States standard population, for the United States (Surveillance, Epidemiology, and End Results (SEER), 2018-2022), Ohio (2017-2021), and Montgomery County, Ohio (2018-2022), by age group. Teal bars represent all ages; brown bars represent women aged 50 years and older; and gold bars represent women aged 65 years and older. Error bars indicate 95% CIs. The rate for Ohio women aged 65 years and older was not reported separately. Sources: National Cancer Institute SEER Program, State Cancer Profiles, and Ohio Cancer Incidence Surveillance System [22-24]. The figure was generated using Python (version 3) and the Matplotlib plotting library.

Montgomery County, which overlies several of the most heavily contaminated portions of the GMBVA, had an age-adjusted incidence rate of 141.4 per 100,000 women (95% CI: 135.7-147.3) during 2018-2022 [24]. This rate was approximately 7% higher than the Ohio statewide rate and 8% higher than the national SEER benchmark, with nonoverlapping 95% CIs (Figure 1). Age-adjusted incidence rates for the US, Ohio, and Montgomery County are shown in Table 1.

**Table 1:** Age-adjusted invasive female breast cancer incidence rates per 100,000 females, standardized to the 2000 U.S. standard population. N/A: Not available because the rate was not separately reported at the state level; SEER: Surveillance, Epidemiology, and End Results. Sources: National Cancer Institute SEER Program, State Cancer Profiles, and Ohio Cancer Incidence Surveillance System [22-24].

Region	All ages (95% CI)	Women aged ≥50 years (95% CI)	Women aged ≥65 years (95% CI)
United States (SEER, 2018-2022)	130.8 (129.9-131.7)	348.1 (347.4-348.8)	435.5 (434.4-436.6)
Ohio (2017-2021)	132.3 (131.2-133.4)	359.5 (356.0-363.0)	N/A
Montgomery County, Ohio (2018-2022)	141.4 (135.7-147.3)	386.6 (370.2-403.5)	491.9 (465.7-519.2)

The corresponding age-adjusted incidence rate ratios, reported as standardized incidence ratios (SIRs), indicated statistically significant elevations. The SIR versus the US was 1.08 (95% CI: 1.04-1.13), and the SIR versus Ohio was 1.07 (95% CI: 1.02-1.11) (Table 2).

**Table 2:** Age-adjusted incidence rate ratios, reported as SIRs, for invasive female breast cancer in Montgomery County, Ohio, relative to the Ohio statewide and U.S. SEER reference populations, by age stratum. SIR = Montgomery County age-adjusted incidence rate ÷ corresponding reference age-adjusted incidence rate, with rates standardized to the 2000 U.S. standard population. The 95% CIs were calculated using log-transformed standard errors derived from the published rate CIs [26,27]. All lower limits of the 95% CIs exceeded 1.0. Ohio did not report a separate rate for women aged ≥65 years. SIR: Standardized incidence ratio; SEER: Surveillance, Epidemiology, and End Results.

Age stratum	Reference population	SIR	95% CI
All ages	United States (SEER, 2018-2022)	1.08	1.04-1.13
All ages	Ohio (2017-2021)	1.07	1.02-1.11
Women aged ≥50 years	United States (SEER, 2018-2022)	1.11	1.06-1.16
Women aged ≥50 years	Ohio (2017-2021)	1.08	1.03-1.12
Women aged ≥65 years	United States (SEER, 2018-2022)	1.13	1.07-1.19

Age-Specific Patterns

Montgomery County had higher incidence rates than both the Ohio and U.S. benchmarks across all examined age groups, and the absolute and relative differences were greater among older women (Figure 1). For women aged 50 years and older, the Montgomery County rate was 386.6 per 100,000 (95% CI: 370.2-403.5), compared with 359.5 in Ohio (95% CI: 356.0-363.0) and 348.1 nationally (95% CI: 347.4-348.8), corresponding to an SIR of 1.08 (95% CI: 1.03-1.12) versus Ohio and 1.11 (95% CI: 1.06-1.16) versus the United States. For women aged 65 years and older, the Montgomery County rate was 491.9 per 100,000 (95% CI: 465.7-519.2), compared with 435.5 nationally (95% CI: 434.4-436.6), yielding an SIR of 1.13 (95% CI: 1.07-1.19). All reported SIRs had lower 95% confidence limits above 1.0, indicating statistically significant elevations relative to the respective reference rates in every age stratum examined (Table 2) [22,24].

Geographic Patterns

**Figure 2:**
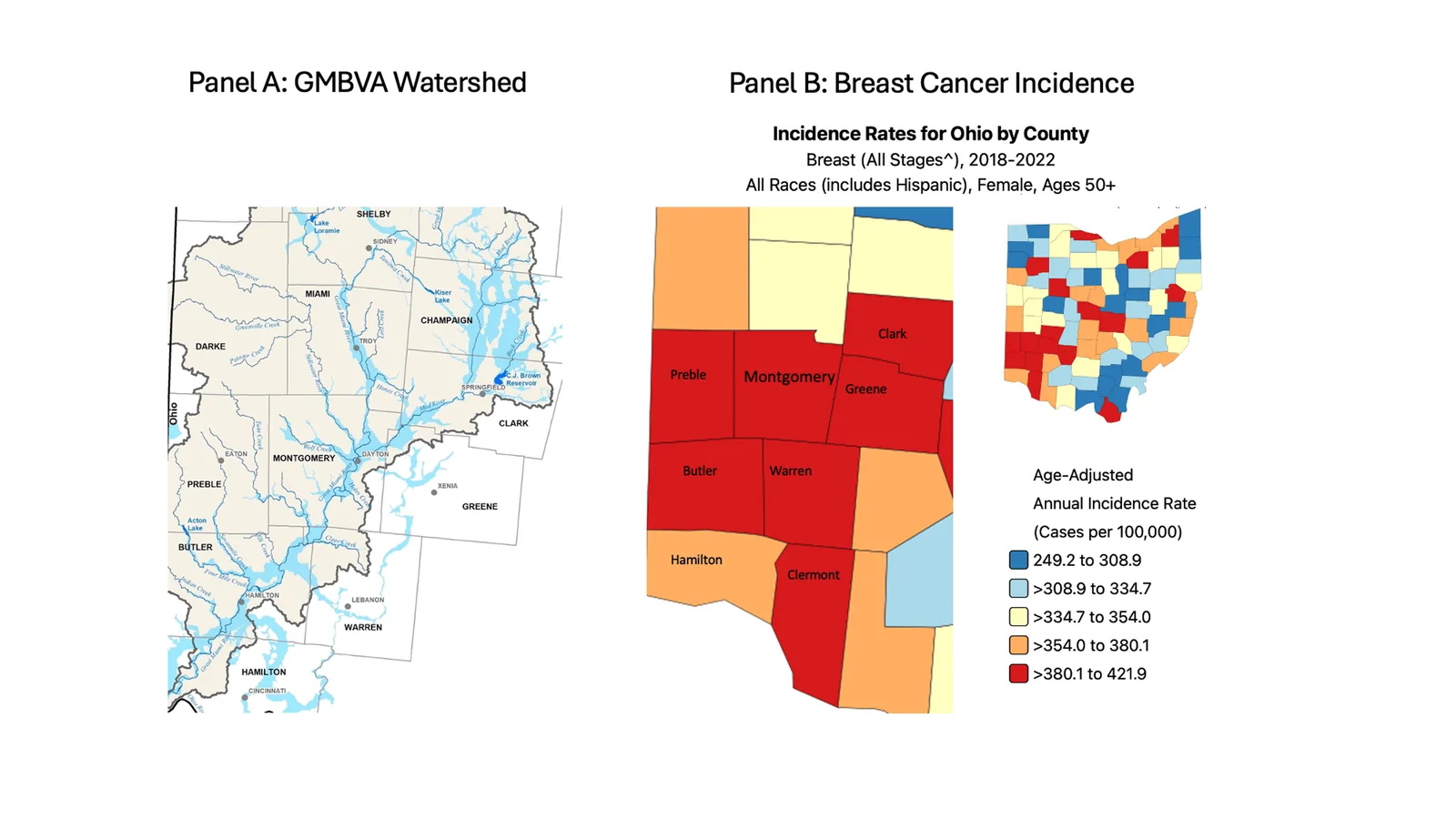
Geographic correspondence between the Great Miami Buried Valley Aquifer (GMBVA) watershed and breast cancer incidence in southwestern Ohio. Panel A: Map of the GMBVA watershed showing the aquifer extent and named tributaries across the core southwestern Ohio counties (Montgomery, Butler, Miami, Greene, Warren, Preble, Darke, Shelby, Champaign, Clark, and Hamilton), with the city of Dayton at the geographic center of the aquifer region (source: Miami Conservancy District [7]). Panel B: Age-adjusted invasive female breast cancer incidence rates per 100,000 among women aged 50 years and older, by Ohio county, for all races (including Hispanic persons) and all stages during 2018-2022. Higher rates are shown in colors ranging from orange to dark red. Montgomery, Butler, Warren, Clark, Preble, and Miami counties fall within the two highest incidence categories (>354.0 to 380.1 and >380.1 per 100,000). Data were obtained from the National Cancer Institute State Cancer Profiles [24]. This composite figure is intended for visual context only and does not represent a formal spatial analysis.

Descriptively, the core counties overlying the more industrialized portions of the aquifer, Montgomery, Butler, Miami, and Greene, appeared to have higher reported incidence rates than less-industrialized counties in the region. County-level age-adjusted invasive female breast cancer incidence rates for all 15 counties overlying the GMBVA, together with a summary of the documented contamination context and the a priori designation of each county, are shown in Table 3. The underlying numeric rates from the 2015-2019 OCISS data are provided in full in Appendix 1. The “core industrialized” versus “comparator” distinction was defined a priori based on the environmental criteria described in the Study design section and was not derived from the incidence data. No quantitative or formal spatial statistical analysis was performed. Publicly available environmental reports indicate that these core counties also contain the GMBVA’s most extensively documented groundwater contamination and active remediation footprints [6-10]. Figure 2 provides a visual, qualitative comparison of the aquifer extent and county-level breast cancer incidence rates among women aged 50 years and older. Figure 2 is intended for visual context only and does not represent a formal spatial analysis; readers are cautioned that the county labels, legend classes, and category cut points reflect the source cartography (National Cancer Institute State Cancer Profiles) rather than a quantitative geostatistical model.

**Table 3:** A priori designation and documented groundwater contamination context for the 15 counties overlying the Great Miami Buried Valley Aquifer. The a priori designation (“core industrialized” versus “comparator”) was specified before the formal comparison of county-level incidence data and was based solely on the environmental criteria described in Study design: the presence of at least one federal Superfund site with a chlorinated-solvent groundwater plume, documented PFAS detections in the USGS 2019-2020 GMBVA well survey [6], and/or listing as an active DoD PFAS-response site. The contamination context was compiled from US EPA Superfund site profiles, the USGS PFAS well survey [6], and US Air Force WPAFB PFAS updates [10,25]. County-level age-adjusted invasive female breast cancer incidence rates for all 15 counties are provided in Appendix 1 (sources: National Cancer Institute State Cancer Profiles and Ohio Cancer Incidence Surveillance System [23,24]). The a priori designation does not coincide with the authors’ clinical catchment area (Montgomery, Greene, and Warren counties), which overlaps with but is not identical to the focal group (see Study design). DoD: US Department of Defense; GMBVA: Great Miami Buried Valley Aquifer; PFAS: Per- and polyfluoroalkyl substances; US EPA: US Environmental Protection Agency; USGS: US Geological Survey; WPAFB: Wright-Patterson Air Force Base; CERCLA, Comprehensive Environmental Response, Compensation, and Liability Act; VOC: Volatile organic compound.

County	A priori designation	Documented contamination context related to the GMBVA
Montgomery	Core industrialized	Multiple Superfund VOC groundwater plumes (Behr Dayton Thermal VOC Plume and Valley Pike VOCs); PFAS-response activities at WPAFB; USGS PFAS detections
Butler	Core industrialized	Industrial legacy in the Hamilton-Fairfield-Middletown corridor; USGS PFAS detections
Miami	Core industrialized	Industrial legacy in the Troy-Piqua corridor; USGS PFAS detections
Greene	Core industrialized	Partially overlaps the WPAFB PFAS-response footprint
Warren	Comparator	Mixed industrial and suburban land use; some documented Superfund and state remediation sites; less extensive PFAS/CERCLA documentation than the core group
Remaining 10 counties (Hamilton, Clark, Champaign, Shelby, Preble, Darke, Logan, Auglaize, Mercer, and Hancock)	Comparator	Generally agricultural or characterized by limited GMBVA-specific Superfund/PFAS documentation. Hamilton County is an exception because of its extensive industrial legacy but is located hydraulically downgradient of the GMBVA core. See Appendix 1 for the contamination context and incidence rates of individual counties.

Groundwater contamination context

PFAS in the Regional Aquifer

**Figure 3:**
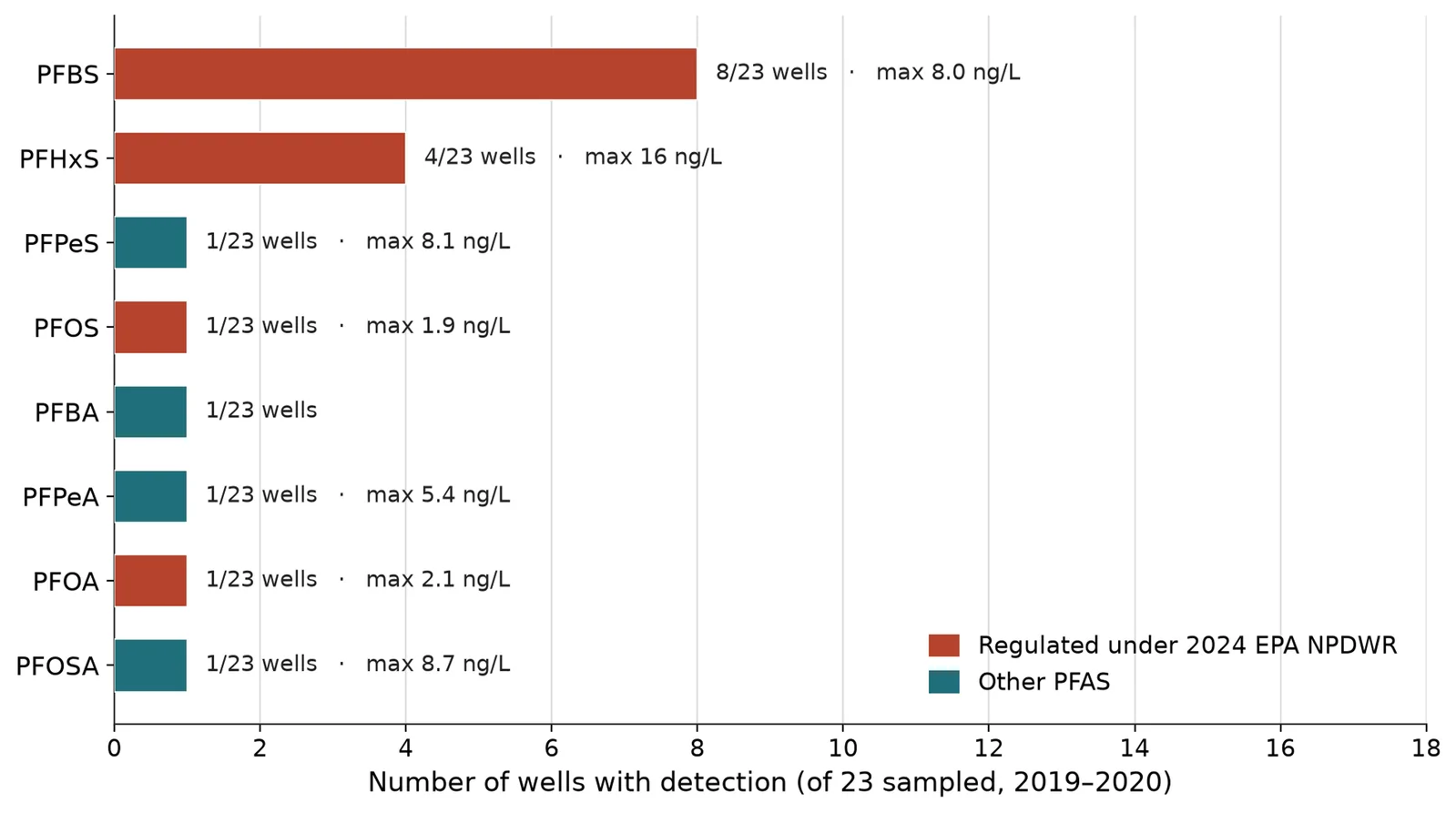
Per- and polyfluoroalkyl substances (PFAS) detected in Great Miami Buried Valley Aquifer wells. Twenty-three wells across nine southwestern Ohio counties were sampled during 2019-2020. The bars show the number of wells in which each analyte was detected; annotations indicate the detection frequency and maximum reported concentration (ng/L). Compounds regulated under the 2024 US Environmental Protection Agency (EPA) National Primary Drinking Water Regulation, perfluorooctanoate (PFOA), perfluorooctanesulfonate (PFOS), perfluorohexanesulfonate (PFHxS), and perfluorobutanesulfonate (PFBS), are shown in orange; other detected PFAS are shown in green. The figure was generated using Python (version 3) and the Matplotlib plotting library.

USGS sampling of 23 GMBVA wells during 2019-2020 detected one or more PFAS compounds in 11 wells (48%), with at least one PFAS detected in each of the nine sampled counties [6]. Of the 24 targeted PFAS compounds, eight were detected at least once. Perfluorobutanesulfonate (PFBS) was the most frequently detected compound (8 of 23 wells; maximum concentration, 8.0 ng/L), followed by perfluorohexanesulfonate (PFHxS) (4 of 23 wells; maximum concentration, 16 ng/L). Perfluorooctanoate (PFOA) and perfluorooctanesulfonate (PFOS) were each detected in one well. The detected PFOA and PFOS concentrations were below their respective 2024 EPA maximum contaminant levels (MCLs) of 4 ng/L but substantially exceeded the EPA’s June 2022 interim health advisory values. However, the maximum PFHxS concentration of 16 ng/L exceeded its 2024 MCL of 10 ng/L. Detections were concentrated in wells associated with predominantly urban land use and oxic redox conditions, consistent with the surface infiltration of PFAS through coarse-grained outwash deposits [6]. Detection patterns by compound are summarized in Figure 3.

PFAS in Finished Water at Wright-Patterson Air Force Base

Finished drinking water at the Wright-Patterson Air Force Base Area B water treatment plant had PFOS, PFOA, and PFHxS concentrations above the numerical values of the 2024 EPA MCLs during 2023-2024 monitoring [10,25]. One Area A plant, equipped with PFAS treatment since 2017, reported all PFAS concentrations below detection limits and currently meets the MCLs.

VOC Contamination at Superfund and Industrial Sites

**Figure 4:**
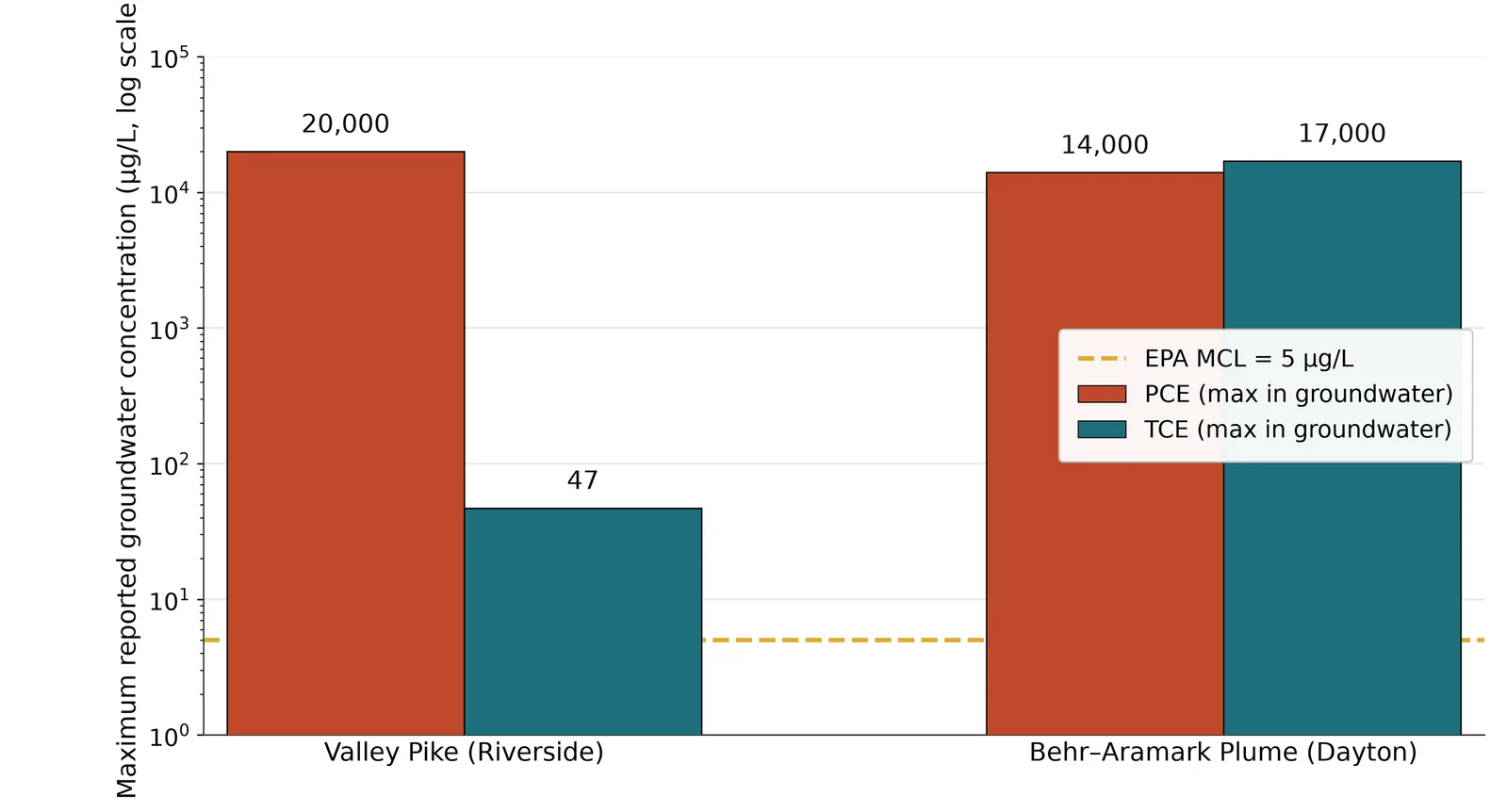
Maximum reported groundwater concentrations of tetrachloroethylene (PCE) and trichloroethylene (TCE) at two Superfund sites in Montgomery County, Ohio, shown on a logarithmic scale (µg/L). The dashed line indicates the US Environmental Protection Agency (EPA) maximum contaminant level (MCL) of 5 µg/L for both PCE and TCE. Data source: US EPA Superfund site profiles. The figure was generated using Python (version 3) and the Matplotlib plotting library.

Several Superfund and active remediation sites within Montgomery County have documented chlorinated-solvent concentrations in groundwater that were orders of magnitude above the federal MCL of 5 µg/L for both TCE and PCE [9,20,21]. At the Valley Pike VOCs Superfund site, PCE concentrations in shallow groundwater reached as high as 20,000 µg/L, approximately 4,000 times the MCL [20]. At the Behr Dayton Thermal VOC Plume Superfund site, monitoring wells reported PCE concentrations of up to 14,000 µg/L and TCE concentrations of up to 17,000 µg/L [21]. Figure 4 summarizes the maximum reported groundwater VOC concentrations at the two Montgomery County VOC Superfund sites relative to the EPA MCLs.

Aggregate Contamination Context

Taken together, these monitoring data document chronic, multidecade releases of solvents and PFAS into a highly permeable sole-source aquifer that supplies the great majority of the region’s drinking water. The geographic distribution of the most heavily contaminated sites, concentrated in Montgomery, Butler, Greene, and Miami counties, descriptively appears to overlap with counties reporting higher age-adjusted breast cancer incidence in southwestern Ohio [24].

## Discussion

Principal findings

This county-level ecological analysis identified a pattern of modestly elevated invasive female breast cancer incidence in southwestern Ohio counties overlying the most contaminated sections of the GMBVA, particularly in Montgomery County. Compared with national benchmarks, Montgomery County exhibited an approximately 8% higher overall incidence (SIR = 1.08; 95% CI: 1.04-1.13), with relative differences appearing somewhat larger in the older age strata (SIRs approximately 1.08-1.13 versus US benchmarks). Age cannot serve as a proxy for cumulative exposure duration or relevant exposure windows in this ecological design because no individual-level data on residential history, years lived in the county, or water source over time were available [22,24,26]. Other core GMBVA counties showed rates above or near the upper range of state and national values, whereas some less industrialized counties had rates closer to or below the averages [24]. The observed descriptive overlap between counties with higher incidence and those with documented historical contamination is notable but cannot support causal inference. Readers should note at the outset that these county-level patterns may also reflect unmeasured differences between counties in mammographic screening utilization, socioeconomic composition, race and ethnicity distribution, urbanicity, reproductive history, obesity prevalence, alcohol consumption, family history of breast cancer, and other established breast cancer risk factors that were not adjusted for in this ecological analysis. These alternative explanations are discussed in more detail under Strengths and limitations.

Comparison with other studies and biological plausibility

Although this study cannot directly attribute elevated incidence to groundwater contamination, prior epidemiologic studies provide biological and epidemiologic plausibility for the hypothesis that long-term exposure to certain VOCs and PFAS may contribute to breast cancer risk in highly exposed populations. The present descriptive, hypothesis-generating findings do not themselves measure individual VOC or PFAS exposure and should not be interpreted as directly supporting the individual-level associations reported in those prior studies. Case-control studies in Cape Cod, Massachusetts, observed an increased risk of breast cancer among women with higher estimated exposure to PCE-contaminated drinking water [11-13]. Investigations at Camp Lejeune have raised concerns about solvent-contaminated water and breast cancer [14]. Meta-analyses of PFAS exposure and breast cancer risk have reported positive but imprecise associations for specific compounds and exposure windows [15,16]. The toxicologic literature indicates that TCE, PCE, and selected long-chain PFAS exhibit carcinogenic, endocrine-disrupting, or immunotoxic properties that are potentially relevant to mammary carcinogenesis [1,3-5].

Taken together, these external data provide context supporting the plausibility of the hypothesis that chronic, low-level exposure to contaminated drinking water may contribute to regional variation in breast cancer incidence, particularly when considered alongside other established risk factors. The magnitude of the excess observed here is modest, and the ecological design precludes the exclusion of alternative explanations, including differences in screening utilization, socioeconomic composition, or unmeasured risk factors.

Public health implications and environmental justice

From a public health perspective, the key contribution of this study is not to demonstrate causality but to illustrate how existing cancer surveillance and environmental monitoring data can be linked to identify communities in which contaminated groundwater may coincide with an elevated cancer burden. The GMBVA region includes urban and suburban populations that have depended on the aquifer for municipal water supplies over many decades, while multiple Superfund and Department of Defense sites remain under active remediation [6-10]. These communities may face overlapping vulnerabilities related to socioeconomic disadvantage, legacy industrial activity, and environmental contamination, raising environmental justice concerns.

Given the substantial methodological limitations, the descriptive patterns do not support specific public health actions, such as prioritizing communities for enhanced screening outreach or risk communication, based on this analysis. Any such recommendations would require individual-level studies with appropriate exposure assessment and control for confounding. Separately from the breast cancer findings, however, continued environmental monitoring, active remediation at Superfund and Department of Defense PFAS-response sites, and transparent reporting of groundwater quality to affected communities remain appropriate public health practices in this region on their own merits, independent of whether a causal link to breast cancer is ever established.

Strengths and limitations

A strength of this study is the use of multiple high-quality, population-based cancer surveillance systems and authoritative environmental monitoring sources, including the USGS PFAS investigation of 23 GMBVA wells across nine counties and EPA Superfund records containing site-specific contamination data.

The study has substantial limitations that severely constrain interpretation and preclude causal inference. First, the purely ecological, county-level design precludes individual-level inference and is susceptible to ecological fallacy. Second, county-level contamination context is an extremely crude proxy that likely results in severe exposure misclassification. The design cannot distinguish municipal water users from private well users or account for point-of-use treatment, residential proximity to specific sources, duration of residence, or individual exposure timing and dose. Third, there was no adjustment whatsoever for confounding. The modest observed differences (approximately 7-13%) could be entirely explained by unmeasured county-level variation in established risk factors, such as race and ethnicity, socioeconomic status, poverty, urbanicity, mammographic screening utilization, obesity, alcohol consumption, reproductive history, or family history. Fourth, breast cancer latency is long, often ranging from 10 to 40 years or more; therefore, current incidence reflects historical exposures. Fifth, the noted geographic correspondence is qualitative and visual only; no formal spatial statistical methods were used. These limitations render the study strictly hypothesis-generating.

Consistent with these limitations, the ecological signal does not show a clear core-versus-comparator gradient. Clark County had the highest single-county age-adjusted incidence rate in the study region (146.7 per 100,000, 2015-2019 OCISS; Appendix 1), despite meeting none of the three a priori environmental criteria and being classified as a comparator. This underscores that unmeasured county-level confounders, including differences in mammographic screening utilization, socioeconomic composition, reproductive history, and undocumented point sources of contamination, may contribute to the observed pattern and cannot be excluded by this ecological design.

Implications for research and practice

Future research should move beyond ecological designs by conducting individual-level cohort or case-control studies that incorporate detailed residential histories, biomarker-based exposure assessments for PFAS (serum) and VOCs, and comprehensive ascertainment of established breast cancer risk factors. Studies evaluating breast cancer subtypes and potential gene-environment interactions could provide further insight into susceptibility. In parallel, continued investment in remediation, transparent reporting of groundwater quality, and collaboration between environmental and public health agencies will be essential to protect communities that rely on contaminated or previously contaminated sole-source aquifer systems.

## Conclusions

This county-level ecological analysis identified descriptive patterns of modestly elevated breast cancer incidence in Montgomery County and some other core industrialized counties overlying the GMBVA. These counties had documented aquifer contamination contexts, federal Superfund designations, USGS PFAS detections, and/or active DoD PFAS-response sites, rather than confirmed individual-level exposures. The small relative excesses (approximately 7-13%) were statistically significant but were unadjusted for confounding and based on a crude county-level exposure proxy. No causal inferences can be drawn, and alternative explanations, including unmeasured differences in screening, socioeconomic factors, or other risk factors, cannot be excluded. The study highlights important methodological challenges in linking aggregated environmental and cancer data and reinforces the need for rigorous individual-level epidemiologic studies with appropriate exposure assessment before any conclusions can be reached about the role of groundwater contamination in regional variation in breast cancer incidence.

## Figures and Tables

**Figure 5:**
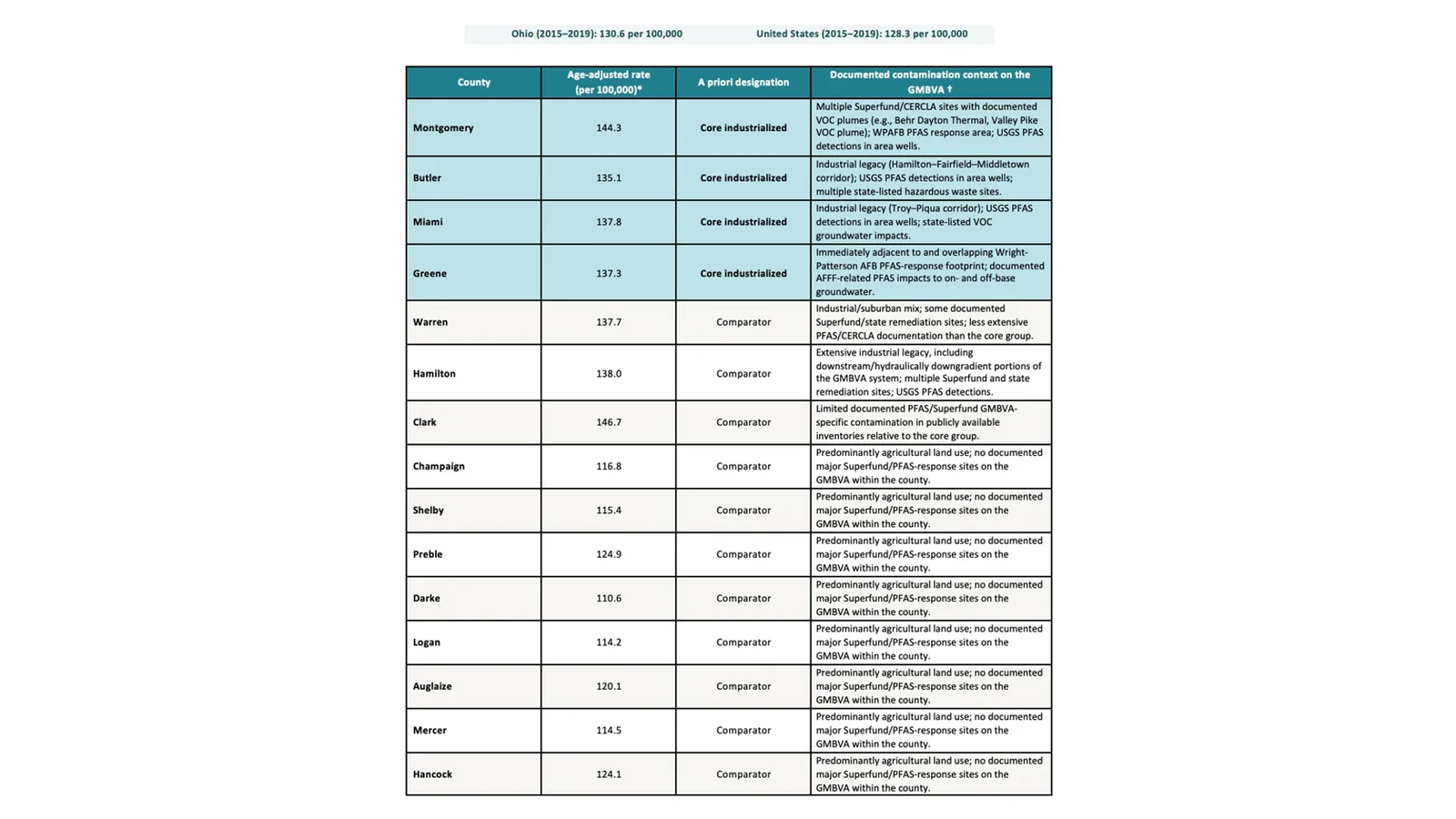
Average annual age-adjusted invasive female breast cancer incidence rates per 100,000 women. * Average annual age-adjusted invasive female breast cancer incidence rate per 100,000 women for all ages, all races/ethnicities combined, and all stages. Age-adjusted to the 2000 U.S. standard population (19 age groups). Reporting period: 2015-2019. Source: Ohio Cancer Incidence Surveillance System (OCISS), Ohio Department of Health, Breast Cancer in Ohio 2022, Figure 3. The comparable 2018-2022 rates published in the ODH 2025 county cancer profiles and the National Cancer Institute State Cancer Profiles (Ohio, 133.0; U.S., 129.8; Montgomery, 141.1; and Hamilton, 141.7) are consistent with the pattern shown here. † Contamination context was compiled from U.S. Environmental Protection Agency Superfund site profiles; the U.S. Geological Survey PFAS well survey (Smalling et al., 2023); U.S. Air Force Wright-Patterson AFB PFAS-response updates; and Ohio EPA remediation records. The a priori designation “Core industrialized” was assigned before any examination of the incidence data to counties meeting one or more of the three protocol-specified criteria (documented Superfund/CERCLA presence overlying the GMBVA; documented Department of Defense PFAS-response footprint overlying the GMBVA; and/or documented USGS PFAS detections in area wells). “Comparator” counties are the remaining 11 GMBVA-overlying counties (Warren, Hamilton, Clark, Champaign, Shelby, Preble, Darke, Logan, Auglaize, Mercer, and Hancock) in which no such contamination has been documented in publicly available inventories as of the study period. ‡ The focal-group designation is based on environmental criteria only and is not congruent with the authors’ clinical catchment area (Montgomery, Greene, and Warren counties), which overlaps with but does not match the focal group. Butler and Miami counties are focal on environmental grounds despite falling outside the practice’s primary catchment, whereas Warren County is treated as a comparator despite being within it.

## Appendices

Appendix 1 

This supplementary figure accompanies Table 3 in the main manuscript and presents the underlying county-level rate data referenced narratively in that table. The rates are average annual age-adjusted invasive female breast cancer incidence rates per 100,000 women, standardized to the 2000 U.S. standard population using 19 age groups, for all races and ethnicities combined, all stages, and the 2015-2019 reporting period. Data source: Ohio Cancer Incidence Surveillance System (OCISS), Ohio Department of Health, Breast Cancer in Ohio 2022, Figure 3. Confirmatory 2018-2022 rates from the National Cancer Institute State Cancer Profiles and ODH 2025 county cancer profiles are consistent with the ranking shown in the supplementary figure (e.g., ODH 2025 rates: Montgomery County, 141.1; Hamilton County, 141.7). The a priori designation (“Core industrialized” versus “Comparator”) was specified in the study protocol before examining the incidence data, using the three criteria stated in Study design: documented Superfund/CERCLA presence overlying the GMBVA, a documented DoD PFAS-response footprint overlying the GMBVA, and/or USGS PFAS detections in area wells.

Notes and limitations

1. This supplementary figure is descriptive. Ecological rates are not evidence of individual-level exposure; they establish only that documented contamination and higher county-level incidence co-occur in the four counties designated a priori as “Core industrialized.”

2. County-level rates are subject to small-count instability in less populous counties (Darke, Logan, Auglaize, Mercer, Preble, Champaign, and Shelby). Point estimates without accompanying confidence intervals should be interpreted cautiously. The OCISS Figure 3 rates are presented using a quartile-based map, and 95% CIs are not published at the county level in the publicly available ODH report. County-specific 95% CIs are available from State Cancer Profiles (SCP) and were consulted when preparing this supplementary figure.

3. Unmeasured county-level confounders, including mammographic screening rates, socioeconomic status, reproductive history (age at first birth and parity), postmenopausal obesity, alcohol use, and family history of breast cancer, were not adjusted for and may contribute to the observed pattern. This is discussed in the main manuscript under Strengths and limitations.

4. Reporting-period alignment: The 2015-2019 OCISS window used in Figure 3 of the Breast Cancer in Ohio 2022 report is used here for county-to-county comparability because it is the only ODH publication that reports rates for all 88 Ohio counties in one standardized product. For Montgomery County only, the main manuscript additionally reports the 2016-2020 and 2017-2021 windows to align with SCP and prior work by our group. These differences in reporting windows do not affect the a priori designation, which was fixed before the analysis.

Data sources

Ohio Department of Health. Breast Cancer in Ohio 2022. Columbus, OH: Ohio Cancer Incidence Surveillance System (OCISS); 2022. Figure 3: Average Annual Age-Adjusted Incidence Rates of Female Breast Cancer per 100,000 by County of Residence, Ohio, 2015-2019.

Ohio Department of Health. County Cancer Profiles (2025 edition; 2018-2022 data): Montgomery County; Hamilton County; Miami County; and companion county profiles.

National Cancer Institute. State Cancer Profiles: Female Breast Cancer, Incidence Rates by County, Ohio (2017-2021 and 2018-2022). statecancerprofiles.cancer.gov.

U.S. Geological Survey. Smalling KL, et al. Per- and polyfluoroalkyl substances (PFAS) in United States tapwater: Comparison of underserved private-well and public-supply exposures and associated health implications. Environ Int. 2023;178:108033.

U.S. Environmental Protection Agency. Superfund site profiles and remediation records for sites overlying the Great Miami Buried Valley Aquifer, including Behr Dayton Thermal VOC Plume, Valley Pike VOCs, and related regional CERCLA sites.

U.S. Air Force. Wright-Patterson AFB PFAS response and site inspection updates. Air Force Civil Engineer Center. 2019-2024.
